# Tubal ectopic pregnancy two years after laparoscopic supracervical hysterectomy

**DOI:** 10.1186/1472-6874-14-69

**Published:** 2014-05-24

**Authors:** Emilia Villegas, Ernesto González-Mesa, María José Benítez, Sergio Luna, Cristina Gómez, Aurelia Marsac, Jesús Jiménez

**Affiliations:** 1 Obstetrics and Gynecology Research Group. IBIMA. Málaga University. School of Medicine. Málaga University Hospital, Málaga, Spain; 2 Obstetrics and Gynecology Department, 12 de Octubre University Hospital, Madrid, Spain

**Keywords:** Ectopic pregnancy, Tubal pregnancy, Hysterectomy, Laparoscopic hysterectomy, Hemoperitoneum, Adnexal mass

## Abstract

**Background:**

Ectopic pregnancy after hysterectomy is a very rare condition, but it must be kept in mind in women with history of hysterectomy who present with abdominal pain and ecographic adnexal heterogeneous images. Since first described by Wendeler in 1895, at least 67 ectopic pregnancies (tubal, ovarian and abdominal) have been described in patients subjected to prior hysterectomy.

**Case presentation:**

We describe the case of a 41-year-old white caucasian woman admitted to the emergency room due to abdominal pain for two days. The ultrasounds scan and the quantification of beta-HCG led to the diagnosis of tubal ectopic pregnancy, although she had been hysterectomized two years before. An emergency laparoscopy was performed for salpingectomy. The pathology report indicated trophoblastic tubal implantation and hematosalpinx.

**Conclusions:**

Ectopic pregnancy is one of the conditions to be considered in the differential diagnosis of abdominal pain in women of child bearing potential, and the absence of the uterus does not rule out its diagnosis.

## Background

Ectopic pregnancy after hysterectomy is very infrequent, yet possible [1]. Since ectopic pregnancy is not suspected in a woman with a history of hysterectomy, the diagnosis may be delayed – with potentially life-threatening risks for the patient.

Ectopic pregnancy is one of the conditions to be considered in the differential diagnosis of abdominal pain in reproductive-aged women. However, absence of the uterus does not rule out a possible ectopic pregnancy. Since first decribed by Wendeler [1] in 1895, 67 ectopic pregnancies (tubal, ovarian and abdominal) have been described in patients subjected to prior hysterectomy. Only one of them has been reported after laparoscopic supracervical hysterectomy (LSH) [2].

After the review articles published by Filstra [3] and Saad Aldin [4], four cases of late ectopic pregnancies in post-hysterectomy patients have been reported, most of them after emergency postpartum hysterectomy. Table 1 shows the main characteristics of these cases [5-8].

**Table 1:** **New cases of late-presentation ectopic pregnancies after hysterectomy **[5]**-**[8]

**Author**	**Year published**	**Type of hysterectomy**	**Time to diagnosis**
Anupama	2012	Emergency supracervical postpartum hysterectomy	11 years
Anis	2013	Emergency supracervical postpartum hysterectomy	6 years
Rajoria	2013	Emergency total postpartum hysterectomy	10 years
Cook	2014	Total abdominal hysterectomy	2 years

The initial clinical symptoms are nonspecific. This fact, and the low clinical suspicion, cause delays in establishing the diagnosis. Most cases present with abdominal or pelvic pain, possibly accompanied by nausea and vomiting. In a few cases vaginal bleeding was the first symptom. There have also been reports of mastalgia, fever, dyspareunia, diarrhea or malaise as first manifestation [9]. The delay in establishing the diagnosis can lead to rupture of the ectopic pregnancy, with an acute and potentially life-threatening acute abdomen. In this context, the mortality rate associated with ectopic pregnancy following hysterectomy is much greater than in patients with ectopic pregnancy and an intact uterus [9]. There are two forms of presentation: early and late. Early presentation comprises ectopic pregnancy in the first weeks or months after hysterectomy. In these cases fertilization had already occurred, or semen was present in the internal genital tract before hysterectomy was performed, thus allowing fertilization of the egg and subsequent ectopic implantation [10].

In turn, the described time interval from hysterectomy to late ectopic pregnancy is between four months and 12 years [11]. The underlying causes appear to be related to three different processes. One mechanism is the presence of fistular tracts between the vaginal dome and the peritoneum, or between the vaginal dome and the Fallopian tube. Such fistulization in turn can be favored by the presence of hematomas or pelvic infections in the region of the vaginal dome [5,12]. A second mechanism that could favor ectopic pregnancy is Fallopian tube prolapse into the vagina, thereby creating a vaginal-tubal communication. Such prolapse could be more common in vaginal hysterectomies, in which there is no direct control of adnexal localization, compared with other types of hysterectom [3]. Lastly, in the case of supracervical hysterectomies, the persistence of cervical permeabilization could facilitate the passage of sperm into the peritoneal cavity.

Early presentations, could be prevented with adequate contraception before hystrerectomy, or scheduling surgery to avoid the luteal phase [13]. In late presentations, the key to prevention is the quality of the hysterectomy technique performed. In this context, the frequency of ectopic pregnancies could be reduced by careful vaginal cuff sealing, keeping the adnexal areas at a distance, and covering with peritoneum. Likewise, as it has been mentioned, avoiding pelvic hematomas and infections reduces the incidence of fistular tracts. Thus, using a correct surgical technique, with careful asepsis and hemostasis, is important for reducing the frequency of this type of pathology. In cases of persistence of the cervix, cauterization of the canal with the purpose of preventing the passage of sperm has been found to be ineffective [10]. However, covering the cervical stump with peritoneum could improve isolation.

In late presentations, the type of hysterectomy performed influences the possible occurrence of ectopic pregnancy, being more frequent after vaginal hysterectomy [3,14] and after emergency postpartum hysterectomy. After vaginal hysterectomy adnexal regions remain very close to vaginal cuff. On the other hand, emergency postpartum hysterectmy is usually performed after dilatation of the cervix and it may contribute to some persistent comunication between the vagina and the peritoneal cavity. Ectopic pregnancy after LSH, as in our patient, is less frequent. Only one case has been previously reported [2].

We report the ocurrence of one case after more than two thousands LSH in our Hospital in the last 10 years, which is a really low incidence. We do not know why the ectopic pregnancy appeared in this patient, but probably her fallopian tubes remained very close to the cervical stump due to fibrosis and adhesions paradoxically caused by previous intra-cesarean sterilization. At present when fallopian tubes remain very close to the cervical stump after supracervical hysterectomy, we electrocoagulate a large portion of both tubes including proximal and distal ends, to prevent eggs from being fertilize.

Ectopic pregnancy must be suspected in hysterectomized patients presenting with abdominal pain or genital bleeding, since a rapid diagnosis is of vital importance [12]. This possibility must be kept in mind when establishing a differential diagnosis, even though the prevalence is very low.

Surgery is the required treatment in all cases. Conservative management with methotrexate is possible, but does not avoid future recurrences; as a result, it cannot be regarded as the treatment of choice [15]. Regarding the type of surgery, removal of the ectopic pregnancy with bilateral salpingectomy is indicated. There have been reports of pregnancies to term, though this is truly life-threatening for the patient [16].

In cases presenting a fistular tract, it should be corrected, and isolation of the remaining cervical stump should be increased in cases of supracervical hysterectomy [17]. The surgical approach can be laparoscopic or using a laparotomy, depending on the urgency of the case and the skill of the surgeon. The vaginal route has also been described, not as a first choice but only in hemodynamically stable cases with a firmly established diagnosis. In these cases, the absence of the uterus facilitates rapid access to the adnexal regions via the vaginal route [18].

## Case presentation

**Figure 1:**
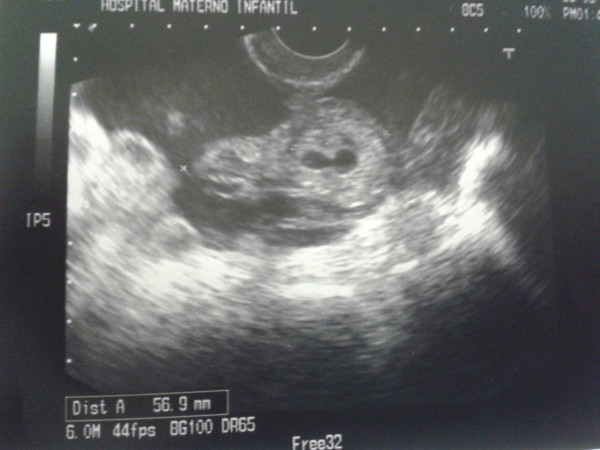
**Transvaginal scan. **Tubal ectopic pregnancy and hemoperitoneum.

**Figure 2:**
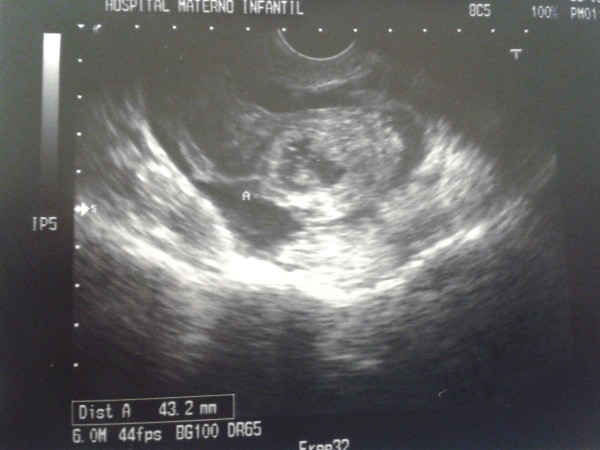
**Transvaginal scan. **Embryonic structures in tubal ectopic pregnancy.

We describe the case of a 41-year-old white caucasian woman seen in the emergency room due to abdominal pain for the last two days. The pain had appeared suddenly and intensely. Her personal history revealed arterial hypertension and chronic venous insufficiency. The gynecological-obstetric antecedents comprised two pregnancies (with cesarean section), with bilateral tubal block performed on occasion of the last pregnancy. In 2011 the patient underwent LSH due to the presence of a polymyomatous uterus. Surgery was uncomplicated and cervical canal was coagulated to avoid any kind of periodical bleeding.Upon admission, the patient was hemodynamically stable, though the pain was described as being very intense. The gynecological exploration proved particularly painful. No masses were palpable in the adnexal regions. The abdomen proved tender, with localized peritoneal irritation in the hypogastric region. Abdominal and transvaginal ultrasound was performed due to the lack of specificity of the symptoms. An echographic heterogeneous, cystic image with hyperechogenic reinforcement was observed in the left adnexal region, suggesting tubal ectopic pregnancy (Figure 1). Embryonic structures were visualized (Figure 2). Given the strong clinical suspicion, serum beta-HCG was determined, yielding a value of 10,259 mU/ml, which consolidated the diagnosis.

Emergency laparoscopy revealed an ectopic pregnancy in the left tubal region, together with hemoperitoneum (500 ml). In view of this finding, which confirmed the diagnosis of ectopic pregnancy, we performed bilateral salpingectomy, with the preservation of both ovaries and sutured the cervical stump to seal the fistula tract. The postoperative course proved normal, with discharge on the fourth day, followed by outpatient controls. The pathology report confirmed the presence of chorionic villi, trophoblastic tubal implantation and hematosalpinx.

## Conclusions

Ectopic pregnancy after any type of hysterectomy is very infrequent, yet possible. It must be kept in mind when establishing a differential diagnosis in all reproductive-aged women with intact ovaries presenting with abdominal pain, since a delay in diagnosis could prove life-threatening for the patient. A rapid diagnosis and correct surgical management reduce mortality and contribute to prevent recurrences. On the other hand, closing the remaining cervical stump with peritonisation in supracervical hysterectomy (or vaginal cuff in vaginal hysterectomy) and removal of Fallopian tubes at the time of hysterectomy might reduce the risk of post-hysterectomy ectopic pregnancy.

## Consent

Written informed consent was obtained from the patient for publication of this case report and any accompanying images. A copy of the written consent is available for review by the Editor of this journal.

## Abbreviations

HCG: Human chorionic gonadotropin; LSH: Laparoscopic supracervical hysterectomy.

## Competing interests

The authors declare that they have no competing interests.

## Authors’ contributions

MJB, CG, JJ and EGM performed the diagnosis and wrote the background and the case report. EV, SL and AM performed the surgery and monitored postoperative course. All authors read and approved the final manuscript.

## Authors’ information

Emilia Villegas and Ernesto González-Mesa are first authors.

## Pre-publication history

The pre-publication history for this paper can be accessed here:

http://www.biomedcentral.com/1472-6874/14/69/prepub
